# Maintenance of Bodily Expressions Modulates Functional Connectivity Between Prefrontal Cortex and Extrastriate Body Area During Working Memory Processing

**DOI:** 10.3390/brainsci14121172

**Published:** 2024-11-22

**Authors:** Jie Ren, Mingming Zhang, Shuaicheng Liu, Weiqi He, Wenbo Luo

**Affiliations:** 1 Key Laboratory of Brain and Cognitive Neuroscience, Liaoning Province, Dalian 116029, China; renjie_lnnu@163.com (J.R.); zmm1001psy@lnnu.edu.cn (M.Z.); lazarusliu@163.com (S.L.); hewq@lnnu.edu.cn (W.H.); 2 Research Center of Brain and Cognitive Neuroscience, Liaoning Normal University, Dalian 116029, China

**Keywords:** bodily expression, visual working memory, delay period, PFC, EBA

## Abstract

**Background/Objectives:** As a form of visual input, bodily expressions can be maintained and manipulated in visual working memory (VWM) over a short period of time. While the prefrontal cortex (PFC) plays an indispensable role in top-down control, it remains largely unclear whether this region also modulates the VWM storage of bodily expressions during a delay period. Therefore, the two primary goals of this study were to examine whether the emotional bodies would elicit heightened brain activity among areas such as the PFC and extrastriate body area (EBA) and whether the emotional effects subsequently modulate the functional connectivity patterns for active maintenance during delay periods. **Methods:** During functional magnetic resonance imaging (fMRI) scanning, participants performed a delayed-response task in which they were instructed to view and maintain a body stimulus in working memory before emotion categorization (happiness, anger, and neutral). If processing happy and angry bodies consume increased cognitive demands, stronger PFC activation and its functional connectivity with perceptual areas would be observed. **Results:** Results based on univariate and multivariate analyses conducted on the data collected during stimulus presentation revealed an enhanced processing of the left PFC and left EBA. Importantly, subsequent functional connectivity analyses performed on delayed-period data using a psychophysiological interaction model indicated that functional connectivity between the PFC and EBA increases for happy and angry bodies compared to neutral bodies. **Conclusions:** The emotion-modulated coupling between the PFC and EBA during maintenance deepens our understanding of the functional organization underlying the VWM processing of bodily information.

## 1. Introduction

Humans possess a remarkable ability to actively maintain and manipulate visual inputs in visual working memory (VWM). This ability facilitates the construction of coherent and continuous representations, enabling the understanding of emotional messages from bodily expressions. Current research widely acknowledges that the critical regions involved in VWM processes encompass the prefrontal cortex (PFC), supplementary motor area (SMA), posterior parietal cortex (PPC), anterior insula (AI), and other higher-order visual areas [1,2,3,4]. These regions within the fronto-parietal network play indispensable roles. However, whether any individual region is sufficient to facilitate the active storage of memory content remains an ongoing subject of debate.

Neural activity within the PFC region is closely linked to VWM storage [5]. Studies focusing on facial expressions have revealed increased PFC activity during a delay period when storing contents such as emotion and identity [6,7,8]. However, there remains an ongoing debate regarding the specific role of the PFC not in information storage but in cognitive control [9,10]. Specifically, PFC activity may reflect a broad range of task variables that do not link directly to the to-be-remembered contents. For example, Rigotti and colleagues found that PFC activity is tuned to mixtures of multiple task-related variables, suggesting that PFC representations exhibit selectivity for high-dimensional information [11]. Further studies indicated that perceptual representations (multi-voxel patterns) are maintained within distributed regions such as the PPC and higher-order visual areas (e.g., facial perception in fusiform gyrus), while the PFC is primarily associated with executive or top-down control over these regions [10,12]. With the accumulation of evidence both in favor of and against the distributed account, it appears to converge on a perspective that information can be flexibly stored [13,14]. Therefore, whether the PFC is also sensitive to directly storing perceptual representations related to bodily expressions during VWM storage needs further exploration.

This distributed storage model also supports the processing of bodily expressions, especially in higher-order visual areas [15]. For instance, it is widely acknowledged that two primary regions demonstrate selectivity for human bodies, the extrastriate body area (EBA) and the anterior fusiform body area (FBA). Both of them are sensitive to the emotional perception of bodily expressions. For example, the perception of happy [16] and angry bodies [17] elicits heightened activation in these regions. Employing multivariate pattern analysis (MVPA), it has been observed that the EBA engages in decoding mid-level features associated with specific body parts to a greater extent than the FBA [18,19]. The perception of dynamic bodily actions or motions is more closely associated with information decoding by the posterior superior temporal sulcus (pSTS) [20,21]. Given that these regions may be sensitive to decoding one or more features during the VWM storage of bodily expressions, this raises questions regarding potential interactions or connections among them. Bodily emotions are expected to enhance the functional connectivity between the PFC and some of these higher-order visual cortices. Although a resting-state fMRI study did not reveal significant functional connectivity between the PFC and EBA [22], further investigation is needed to understand how bodily emotions in VWM storage influence this connection.

This study aims to achieve two primary objectives. The first is to investigate the influence of emotional types of bodily expressions on whole body activity and corresponding representations during the initial stage of stimulus presentation. To this end, both univariate and multivariate analyses were utilized [23,24,25,26]. The purpose of these methods is to examine whether bodily expressions not only enhance neural activity in body-selective regions (EBA and FBA) but also modulate the decoding strategies within these areas. Furthermore, the MVPA method provides an assessment of whether the PFC is involved in storing specific features from bodily expressions. If the PFC exhibits insensitivity to content-specific coding, it should not be among those brain regions where classification differences in bodily expressions are observed. The second objective is to evaluate functional connectivity during the delayed stage following stimulus offset among brain regions involved in the prior presentation stage. The psychophysiological interaction (PPI) methodology was employed. It is commonly utilized to assess whether the functional connectivity strength between target brain regions or regions of interest (ROIs) is influenced by the independent variables under investigation [27]. If the activation or decoding areas are functionally disconnected for maintaining bodily expression, there should not be significant functional couplings between body areas (EBA, FBA) and cognitive control regions (bilateral PFC) for happiness and anger compared to neutral bodies during the later stage [28]. This hypothesis is crucial for determining whether the VWM storage of bodily expressions persists beyond stimulus presentation.

Therefore, the experimental procedures were designed to encompass the three following stages: (1) initial stimulus presentation, (2) a delayed period of blank screen, and (3) a response stage for emotion categorization. The stimulus materials encompassed two distinct emotions along with a neutral expression. Emotion perception required the explicit categorization of bodily expressions through forced choice among three alternatives, while body orientation facilitated the implicit recognition of physical attributes. This additional orientation variable was to eliminate cognitive biases in recognizing bodily emotions due to the observational perspective. Regarding the anticipated results, we expect that bodily expressions will initially be decoded in the EBA and PFC and subsequently maintained for a period through enhanced functional connectivity between the two or more regions during delayed-period processing.

## 2. Materials and Methods

### 2.1. Participants

In the current study, 30 participants were recruited (16 females; mean age: 22.1 years, age range: 19–26 years; all right-handed). All participants had normal or corrected-to-normal vision and no documented history of psychiatric or neurological disorders. The experiment received approval from the Ethical Committee at Liaoning Normal University and was conducted in accordance with the Declaration of Helsinki. Informed written consent was obtained from all participants prior to the experiment, and they were financially compensated for their participation.

### 2.2. Stimuli and Experimental Procedure

The bodies were selected from the Bochum Emotional Stimulus Set (BESST) [29]. We chose 24 body images representing four actors (two females; actor IDs: 36, 39, 57, and 61) with two orientations (45° averted view and 0° frontal view). Each actor expressed three different emotional expressions (anger, happiness, neutral). The image size was adjusted to 350 × 500 pixels.

**Figure 1:**
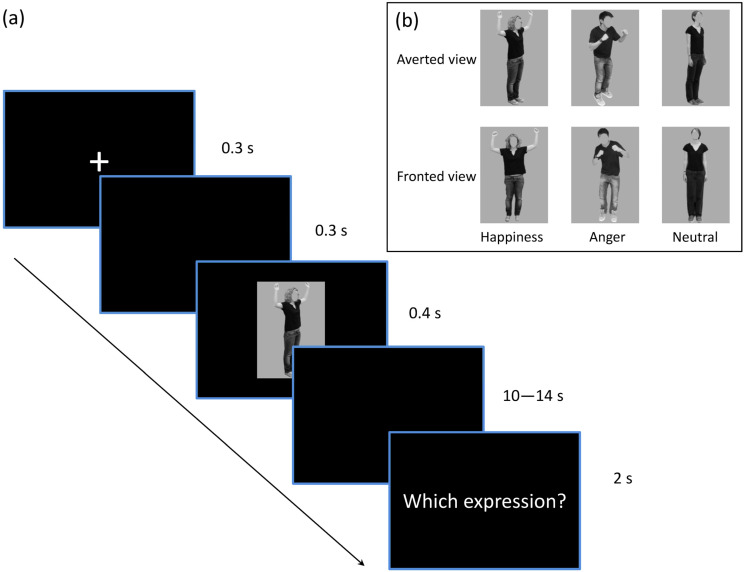
The experimental design. (**a**) The procedure for a single trial. The target stimulus was presented for 400 milliseconds following a fixation screen, succeeded by a blank screen for either 10, 12, or 14 s. Participants were asked to categorize their emotions until the final response interface appeared. (**b**) Examples of experimental stimuli: six body conditions of the factors of emotion (happiness, anger, neutral) and orientation (averted view, fronted view).

The whole experimental procedure was conducted for each participant during MRI scanning. We employed an event-related design consisting of six independent scanning runs each comprising 24 trials (see Figure 1). Each trial began with a fixation cross presented at the center of the viewing screen for 300 ms, followed by a blank screen for another 300 ms. Each stimulus subtended a visual angle of 8.9° × 12.7° and was displayed for 400 ms. Participants were instructed to observe and hold the body stimulus in memory during subsequent blank screens lasting either 10 s, 12 s, or 14 s (in pseudo-random order). Following this interval, a response screen appeared for 2 s displaying three options—happiness, anger, and neutral—corresponding, respectively, to the index finger, middle finger, and ring finger responses. The order of response options was counterbalanced across participants. Each body image appeared only once per run.

### 2.3. fMRI Data Acquisition

Participants underwent MRI scanning using a 3-Tesla MRI scanner (Discovery MR750 3.0T, GE Healthcare, Chicago, IL, USA) equipped with an 8-channel head coil. The implementation of padding and earplugs effectively minimized head movements and attenuated scanner noise. A mirror mounted on the head coil allowed participants to view body stimuli projected via a video projector (refresh rate: 60 Hz; screen resolution: 1280 × 1024) onto the center of a screen located at the rear of the scanner bore. The distance from the screen to the eye mirror was maintained at 80 cm, while the distance from participants’ eyes to the center of the mirror was set at 11 cm. The BOLD signals were measured at a resolution of 3 × 3 × 3 mm^3^ with a gradient echo planar imaging sequence (TR: 2 s; TE: 29 ms; FOV: 192 × 192 mm^2^; matrix: 64 × 64; flip angle: 90°; slice thickness: 3 mm; slices: 43 without gaps; slice orientation: oblique). After removing the volumes from the dummy scan, we collected 181 functional volumes for each run. Additionally, a high-resolution 3D structural dataset (T1-weighted MPRAGE sequence; 1 × 1 × 1 mm^3^ resolution; TR: 6.9 s; TE: 3 ms; flip angle: 8°; 176 slices) was obtained for each participant.

### 2.4. fMRI Data Preprocessing

The fMRI data were preprocessed using the preprocessing pipelines of the CONN toolbox 22.a (http://www.conn-toolbox.org, accessed on 2 June 2023 [30]). The raw data were adjusted to account for differences in acquisition times between slices of each brain volume and subsequently realigned within runs. Potential outliers were identified through the ART algorithm with a framewise displacement above 0.9 mm or global signal changes surpassing five standard deviations. Following indirect segmentation and normalization, anatomical data—segmented into gray matter, white matter, and cerebrospinal fluid tissue classes—were normalized to a standard MNI space, while functional data were co-registered using the same deformation of each participant’s anatomical image. Four participants exhibiting excessive head motion and significant global signal fluctuations were excluded from analysis, resulting in a final sample size of 26 participants. The functional data underwent spatial smoothing with a Gaussian kernel filter set at 4 mm full width at half maximum.

### 2.5. Behavioral Analysis

The repeated-measures ANOVA with a 2 (bodily orientations: averted view, fronted view) × 3 (bodily expressions: happiness, anger, neutral) within-subjects design was performed to test the accuracy of the bodily expressions. The *p*-values were adjusted using the Greenhouse–Geisser method when necessary. Bonferroni correction was applied for post hoc multiple comparisons.

### 2.6. Univariate Analysis

Utilizing SPM12 (http://www.fil.ion.ucl.ac.uk/spm/, accessed on 13 January 2020), a participant-level general linear model (GLM) was constructed in which each condition (averted happiness, averted anger, averted neutral, fronted happiness, fronted anger, fronted neutral) was represented by a square wave of the same duration as the body picture presentation, convolved with the canonical hemodynamic response function. The six runs of the fMRI data were concatenated for each participant. Nuisance regressors included (a) the fixation cross (0.3 s) and the blank epochs (10–14 s) and response periods (2 s) in each trial; (b) the 6 head motion parameters (x, y, z, roll, pitch, and yaw) from the realignment procedure; and (c) the whole trials with error response. To examine the observed effects across participants, the individual brain maps (one contrast image for each condition) were combined for a group-level random effects GLM analysis. To assess the effects and interaction between two factors (orientation and emotion), a two-way repeated measures ANOVA was performed using the SPM’s statistical tools. The results are reported at an uncorrected voxel-based threshold of *p* < 0.001 and were cluster corrected at *q*(FDR) = 0.05.

### 2.7. Multivariate Analysis

Initially, the BOLD time course of each voxel from unsmoothed data was segmented into individual trials, with the temporal window (epoch) defined to match the duration of the body stimulus, resulting in 24 trials per run (144 in total). Subsequently, a within-run GLM comprising six conditions was established to model and estimate each trial for every voxel. The fixation cross and response periods were also incorporated as nuisance regressors. One-sample t-tests were conducted on each epoch of body presentation for every voxel from a single trial, and the statistical t-values were utilized as features in the classifier.

We employed a whole-brain searchlight method, implemented using scripts from the CoSMoMVPA toolbox (http://www.cosmomvpa.org, accessed on 10 Oct 2021 [31]). The t-value brain map was divided into spheres of searchlights for each trial and participant. A Naïve Bayes classifier was fitted within each searchlight. Each spherical searchlight was defined by a radius of four voxels along with its neighboring voxels. The mean decoding accuracy for each sphere was computed through a leave-one-run-out procedure (6-fold cross-validation). Prediction accuracy was assessed by testing the trained classifier on the left-out test data, with the resulting accuracy assigned to the central voxel. To investigate whether the EBA, FBA and PFC decoded information relevant to emotion categorization, the Naïve Bayes classifier was trained to predict three conditional contrasts, namely (1) happy bodies vs. neutral bodies, (2) angry faces vs. neutral faces, and (3) inter-emotion differences (48 trials for each emotional condition).

For the group-level analysis, we applied spatial smoothing with a 3 mm kernel full width at half maximum (FWHM) to the resulting maps. Each decoding map represented classification accuracies relative to chance levels. Statistical inference was conducted using a two-tailed *t*-test against zero across participants. The results were reported using the same threshold as in univariate analyses. For visualization purposes, statistical maps were projected onto cortical surfaces utilizing Surf Ice (https://www.nitrc.org/projects/surfice, accessed on 29 Nov 2021).

### 2.8. Functional Connectivity Analysis: gPPI

We further conducted a generalized psychophysiological interaction (gPPI) analysis to evaluate whether functional connectivity (FC) between the PFC and EBA increased for maintaining emotional information. It was implemented using the unsmoothed volumes processed in the preceding univariate analysis. To exclude the interference of non-neural signals, an anatomic component-based noise correction (aCompCor) strategy was adopted, which involved removing confounding effects through linear regression from white matter, cerebrospinal fluid (CSF), six realignment parameters, and their corresponding scrubbing parameters as noise components [32]. The image volumes were band-pass filtered at 0.008–1 Hz. The gPPI model was specified according to the following equation:

y = *β_0_* + *β_1_* × R + *β_2_*AvertedHB + *β_3_*AvertedAB + *β_4_*AvertedNB + *β_5_*FrontalHB + *β_6_*FrontalAB + *β_7_*FrontalNB + *β_8_*AvertedHB × R + *β_9_*AvertedAB × R + *β_10_*AvertedNB × R + *β_11_*FrontalHB × R + *β_12_*FrontalAB × R + *β_13_*FrontalAB × R,

where R is the averaged time series of the ROI; AvertedHB, AvertedAB, AvertedNB, FrontalHB, FrontalAB, and FrontalNB are the psychological regressors (maintenance periods) representing the six conditions; and AvertedHB × R, AvertedAB × R, AvertedNB × R, FrontalHB × R, FrontalAB × R, FrontalAB × R are the psychophysiological interactions between the psychological regressors and the averaged time series of each ROI.

Whereas the univariate and multivariate analyses were performed based on the period of stimulus presentation, the connectivity analysis focused on the period of maintaining stimulus. All of the three analyses utilized specific assets of the experimental design. The univariate and multivariate analyses targeted the body representing events related to the visual perception of bodily expressions. In contrast, the connectivity analysis enhanced power by extending events across longer maintaining periods [33]. As implemented in CONN, psychological regressors were convolved with the hemodynamic response function (HRF), and psychophysiological interactions were modeled using raw BOLD-level signals.

The ROI-to-ROI analysis was directly conducted, in which we estimated the interaction terms (*β_8_* to *β_13_*) by using the regression coefficients to measure the connectivity of an averaged time series in every target ROI to each of the left-out seed ROIs (ROI selection in the next paragraph). Then, these interactions were subjected to a second-level random effects analysis. To explore if the connectivity between any two ROIs had any significant effect of interest, F tests were conducted jointly on all the interaction terms separately for two groups of ROIs. Spatial statistics were obtained using threshold-free cluster enhancement (TFCE) [34], whose contrasts were investigated with a *p*-FWE-corrected connection threshold of *p* < 0.05.

**Figure 2:**
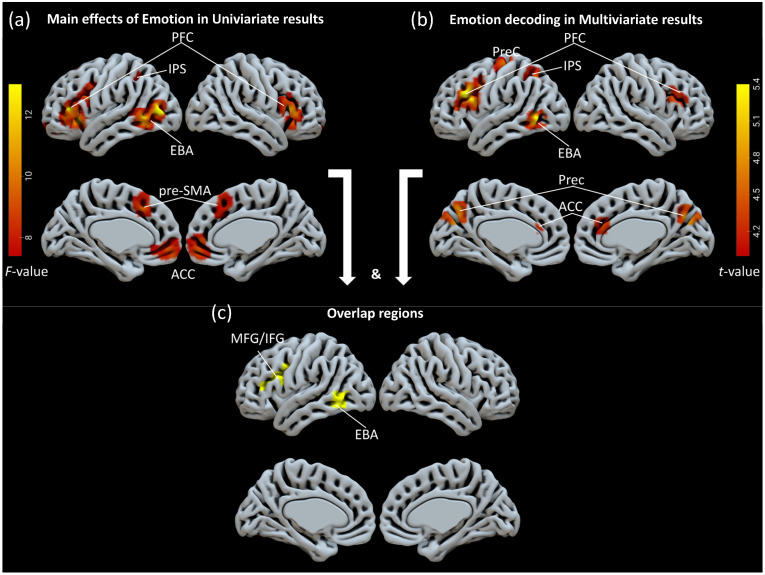
(**a**) The brain activation of the main effect of emotion. The whole-brain map is revealed through univariate analysis. (**b**) The decoding accuracy of the three classifications of emotion. The whole-brain map is revealed by multivariate analysis. (**c**) Overlapped regions between (**a**,**b**). Highlighted in pure yellow, the map shows the extent of the overlap for comparison between the analytical methods. Abbreviations: PFC = prefrontal cortex; IPS = intra-parietal cortex; EBA = extrastriate body area; PreC = precentral cortex; Prec = precuneus; pre-SMA = pre-supplementary motor area; ACC = anterior cingulate cortex; MFG/IFG = middle frontal gyrus/inferior frontal gyrus.

The ROIs were created based on the statistical outcomes derived from the main effects of emotion in univariate analyses, as well as from the inter-emotion decoding in multivariate analyses (for significant effects, refer to Figure 2). We conducted separate examinations of associations within two distinct groups, each encompassing all significant clusters identified in either univariate or multivariate results. The ROIs identified through the univariate analysis included regions located in the PFC (l/r; geometric centers at [−43, 24, 9] and [49, 25, 11]), AI [40, 27, −6], ACC [2, 55, −4], pre-SMA [−2, 22, 45], EBA [−50, −61, 0], IPL [−31, −44, 42], and angular gyrus (AG) [40, 27, −6] (a total of eight ROIs). The ROIs determined from the multivariate results comprised those situated in the PFC (l/r; [−46, 19, 27], [50, 21, 26]), ACC [5, 35, 21], IPS [−33, −59, 49], PreC [−33, −20, 65], Precu [−1, −71, 35], and the EBA [−55, −62, −3] (a total of seven ROIs).

## 3. Results

### 3.1. Behavioral Performance

To examine any difference in performance under the two orientations and three emotions, a two-way repeated measures ANOVA was conducted on accuracies. The analysis revealed a significant main effect of emotion (*F* (2, 50) = 20.456, *p* < 0.001, *η_p_*^2^ = 0.45). However, the main effect of orientation did not reach a significant level (*F* (1, 25) = 0.771, *p* = 0.38, *η_p_*^2^ = 0.03). The interaction between the orientation and emotion was also significant (*F* (2, 50) = 5.281, *p* = 0.008, *η_p_*^2^ = 0.174). To break down the interaction, ANOVAs were conducted separately for the fronted view and averted view. For the fronted view condition, the mean accuracy for neutral bodies (0.902 ± 0.023) was significantly higher than the mean accuracy for happy bodies (0.731 ± 0.041; *p* < 0.001) and angry bodies (0.635 ± 0.04; *p* < 0.001). There was no significant difference in the mean accuracy for happy and angry bodies (*p* = 0.066). For the averted view condition, the mean accuracies for neutral bodies (0.836 ± 0.041; *p* = 0.008) and happy bodies (0.799 ± 0.042; *p* = 0.024) was significantly higher than that for angry bodies (0.688 ± 0.039), respectively. There was, however, no significant difference in the mean accuracy for neutral and happy bodies (*p* = 0.708). Together, these statistics reflect a reduced behavioral difference between the emotional and non-emotional expressions in an averted view.

### 3.2. Analysis of Condition Effects at Activation Level

**Figure 3:**
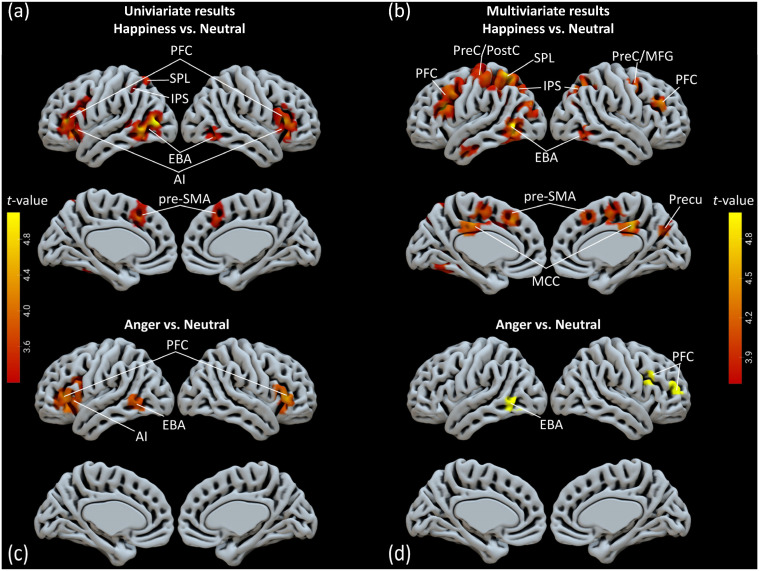
The brain activation shown in the contrast analysis indicates that (**a**) happiness > neutral and (**c**) anger > neutral. Additionally, we assessed the decoding accuracy for the classification between (**b**) happiness and neutral, as well as between (**d**) anger and neutral. Abbreviations: PFC = prefrontal cortex; SPL = superior parietal lobule; IPS = intra-parietal sulcus; EBA = extrastriate body area; AI = anterior insula; pre-SMA = pre-supplementary motor area; PreC/PostC = pre-central gyrus/post-central gyrus; MCC = middle cingulate cortex; Precu = precuneus.

We examined whole-brain activation elicited by the two main factors (orientation: averted view, fronted view; emotion: happiness, anger, neutral) and their interactions. No significant main effects for the orientation factor were found after cluster threshold correction. Significant main effects for the emotion factor were observed in clusters located in the PFC, IPS, and EBA in the left hemisphere and the PFC, AI, and AG in the right hemisphere. The clusters in the pre-SMA and ACC were observed in the medial surface of the brain (Figure 2a, Table 1). The contrast of happy vs. neutral bodies revealed significantly increased activation for happy bodies in the bilateral PFC, pre-SMA, bilateral EBA, and the IPS (Figure 3a, Table 2). The contrast of anger vs. neutral bodies revealed significantly increased activation for angry bodies in the bilateral PFC and left EBA (Figure 3c, Table 2). No significant activation was found for the contrast of happy bodies vs. angry bodies.

### 3.3. Multivariate Decoding of Emotion-Related Information

The three expressions exhibited significant differences in bodily postures as a response to specific emotional contexts. This observation raises the question of whether these variations in posture are correlated with neural decoding primarily occurring in posture-sensitive areas. The whole-brain Naïve Bayes searchlight analysis demonstrated significant above-chance classification of the three emotions in the seven following clusters: the bilateral PFC, left EBA, ACC, Precu, IPS, and PreC (Figure 2b, Table 1). Furthermore, these decoding regions overlapped with activation areas identified through a univariate analysis encompassing 57 voxels in the PFC and 109 voxels in the EBA (Figure 2c). The findings indicated a preference for decoding bodily postures predominantly within the left hemisphere. The precise localization of these two regions suggests functional correlations with the representations of bodily postures, potentially indicating that emotional effects are expressed not only through levels of activation but also via distinct information decoding patterns.

Subsequently, we conducted an analysis comparing happy versus neutral body classifications, which revealed above-chance decoding accuracies within the anterior temporal lobe (ATL), bilateral PFC, central sulcus (CS), bilateral IPS, left superior occipital gyrus (SOG), pre-SMA, middle cingulate cortex (MCC), bilateral PreC, and bilateral EBA (Figure 3b, Table 2). In contrast, the analysis of the angry versus neutral body classification yielded above-chance results specifically localized within the PFC and right EBA (Figure 3d, Table 2).

### 3.4. Functional Connectivity

**Figure 4:**
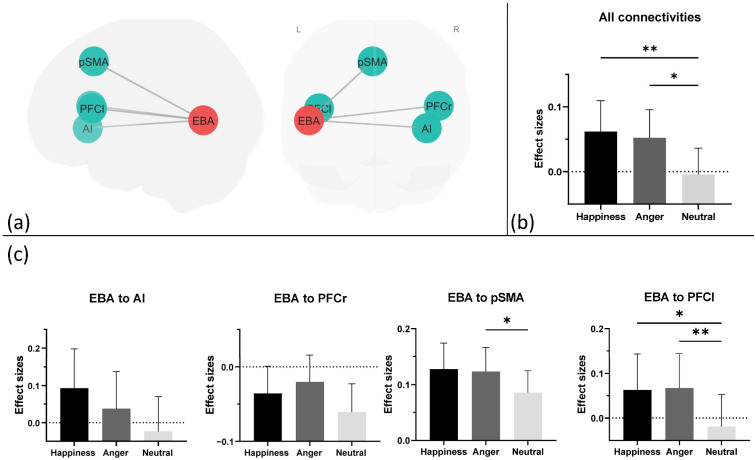
(**a**) A graphical representation of ROI-to-ROI connection results. The corrected connections include five ROIs, namely the left EBA, left and right PFC, pSMA, and AI derived from the univariate activation results. The (**b**) graph presents the statistical outcomes for the mean strength of all significant ROI-to-ROI connections, while (**c**) another graph provides a detailed account of each significant connection. Abbreviations: pSMA = pre-supplementary motor area; PFCl = left prefrontal cortex; PFCr = right prefrontal cortex; AI = anterior insula; EBA = extrastriate body area. *: *p* < 0.05, **: *p* < 0.01.

We further sought to elucidate which specific connections within the area were related to storing bodily expressions during the maintenance period. These connections were investigated separately for the activation regions and for the decoding regions using ROI-to-ROI gPPI analysis. Within the activation regions, a set of four significant functional connections were found according to the main effects of emotion; the left EBA exhibited significant FCs to the pre-SMA, right AI, left PFC and right PFC (Figure 4a). Next, we examined how the emotion effects existed separately in every single connection through a one-way ANOVA statistical analysis (Figure 4c). This revealed significant main effects of emotion on the connection of the left EBA to the pre-SMA (*F* (2, 50) = 5.143, *p* = 0.015, *η_p_*^2^ = 0.171) and the left EBA to the left PFC (*F* (2, 50) = 5.624, *p* = 0.009, *η_p_*^2^ = 0.184). Pairwise comparisons showed that the connection of the left EBA to the pre-SMA demonstrated stronger FCs for angry bodies than neutral bodies (*t* (25) = 2.93, *p* = 0.021). For the EBA to the left PFC, stronger FCs were observed for happy bodies than neutral bodies (*t* (25) = 3.05, *p* = 0.016) and for angry bodies than neutral bodies (*t* (25) = 3.44, *p* = 0.006). Finally, no other significant alterations in FCs were observed within these activation regions or among the predefined decoding-related regions.

## 4. Discussion

In the current study, we investigated the brain regions involved in the initial encoding of emotional bodily expressions and examined the functional connectivity between the regions during a delayed maintenance period in VWM. Our findings revealed three key insights. First, an enhanced processing of emotional bodies was observed in the increased activation and improved decoding accuracy within the left EBA and left PFC. Second, the happy bodies elicited broader activation engagements compared to angry bodies in the left IPS and pre-SMA, while decoding results indicated involvement of the IPS, pre-SMA, as well as both pre- and post-central gyrus. This may be related to the fact that happy bodies involve more exaggerated body movements, which may consume more cognitive resources. Third, both happy and angry bodies strengthened functional connections from the EBA to the bilateral PFC, pre-SMA, and AI, shedding light on how brains integrate bodily posture into VWM storage of emotional information. In subsequent sections, we discuss these results to elucidate where and how these bodily postures are processed through initial decoding followed by later maintenance.

### 4.1. Brain Areas for Initial Processing

Our primary findings indicate that human observers are capable of initially encoding stimuli within three prominent regions (EBA, pre-SMA, and PFC) during a brief presentation. We discuss the roles of the first two regions in this section. Previous research on bodily expression perception has extensively described the selectivity of these regions; the EBA exhibits specificity for body parts or detailed body information [35,36], while the pre-SMA is implicated in action preparation [37,38]. The EBA is a multifaceted region encompassing significant substructures that may encode distinct features of body image [39]. Consistent with the current perspective on feature-specific processing [15,40], responses within these visual regions appear to be sensitive to stimulus attributes. For example, the EBA exhibited enhanced selectivity towards postural features, such as limb contraction [19]. In this study, the overlap between our univariate and multivariate results suggests a comprehensive processing and encoding of bodily features. In the presence of happy or angry expressions, the EBA may facilitate a rapid and preliminary construction of others’ adaptive behavioral patterns [37]. It probably extracts motion features from specific body parts like hands and arms [41], because hands and arms have been demonstrated to play an indispensable role in understanding happy and angry expressions [19,42,43,44]. In addition, our findings regarding hemisphere preferences diverge from some previous studies indicating a right preference [23,45]; instead, our results reveal a preference for the left EBA. However, this lateralization aligns with selective responses to hands rather than other body parts observed in the left EBA [46]. One of our prior works [18] also demonstrated a left hemisphere advantage on decoding bodily information, which requires further experimental validation due to potential differences in stimulus materials or participants.

The pre-SMA is another important ROI, which has been shown to exhibit a tight relationship with motivated voluntary actions and often involved in the selection and preparation of internal motor plans [47]. The pre-SMA may utilize body-related information to comprehend individuals’ intentions [48] and prepare for an appropriate response [49]. Notably, both univariate and multivariate analyses have observed significant results specifically related to happy expressions. This finding is not consistent with the pre-SMA’s inhibitory function for negative emotions. In the current context, Pichon and colleagues (2012) [38] revealed positive correlations with threat-related responses in the pre-SMA and behavioral reaction time (RT) for both fear and anger relative to neutral expressions. Thus, the pre-SMA probably plays a role in suppressing the emotional motor response arising from perceived emotions. However, the pre-SMA was observed in angry bodies in subsequent connections, and considering the correlation results of pre-SMA signals and RT in Pichon’s study [38], it appears that the influence of the pre-SMA may extend into the subsequent maintenance period.

Our findings demonstrate limited FBA activation and its multi-voxel patterns. Both the FBA and EBA are specialized in processing body-related information [50]. However, the perception of the whole body is more closely linked to the FBA than to the EBA. In terms of emotion functions, the prevailing perspective suggests that the EBA encodes mid-level features related to body parts, postures, and position rather than directly contributing to higher-level perceptions such as identity, race, and emotion [19]. These latter functions are believed to be potential roles of the FBA through its connections with other brain regions [36]. This may explain the inclusion of a few FBA results within EBA clusters in the study.

### 4.2. Importance of PFC in Information Storage

Additionally, the prefrontal regions including MFG and IFG are known by cognitive control mechanisms through which they modulate visual perception [28,51]. Is the maintenance period characterized by a deep processing of initial encoding or by the storage of specific information? Our findings primarily support the latter.

In human subjects, sustained BOLD activation in the absence of external input has been considered a marker of working memory storage since the work of Courtney et al. [52]. Our findings of multivariate analyses align with prior research indicating emotional sensitivity in the PFC, which has established the pivotal role of information storage [53,54]. Recent studies using inverted encoding modeling have successfully reconstructed remembered features from activation patterns in the PFC during the delay period [55,56]. They supported the notion of specific information storage. It appears premature to dismiss the role of the PFC in the actual storage of stimulus-specific features during the VWM delay.

In the context of emotion regulation, there exists a faction that advocates for the notion that neurons within the PFC are attuned to nonlinear combinations of various task-relevant dimensions [14,57]. The activation of the PFC is strongly associated with overall task performance or increased demands on targeted items. Hebart and colleagues (2018) [58] clearly demonstrated the general task effect by revealing an augmented sequence of shared variances between MEG and fMRI signals from the prefrontal cortex, providing support for both when and where the effect occurs. One possible reason for the observed effect in modulating emotion processing is that the PFC also harbors adaptable feature representations, such as body-selective neurons, which are recruited when necessary for task performance.

Additionally, our results failed to reveal any connections between the PFC and PPC. An important piece of evidence indicates that fronto-parietal connections are related to Gerstmann’s syndrome (GS) [59]. Patients with GS exhibit deficits in finger recognition and left or right orientation discrimination, which suggests the importance of this connection for the cognition of body postures and actions. Although both the univariate activation and multivariate encoding results showed enhanced SPL effects for happy bodies during the stage of stimulus presentation, no enhanced functional connectivity was observed for such bodies during the subsequent stimulus-absent stage. One explanation is that during this stage, perceptual representations about the location and orientation of the body parts were not VWM-stored as significant task-related information.

### 4.3. Involvement of Functional Connectivity in Maintaining Period

In terms of connections, body expression memorization is directly associated with the EBA and its connection with the bilateral PFC. First, the functional connections between the EBA and IFG are grounded in the structural profile of the arcuate fasciculus (AF), which plays a crucial role in verbal fluency [60] and working memory [61], thereby serving as a component of the language network [62,63]. However, the AF is also engaged in understanding emotional states in others based on facial expression and other cues [64,65], indicating potential physiological foundations for perceiving and memorizing bodily expressions.

Second, consistent with the existing literature in the functional profile [14], our findings also revealed robust functional connections between the PFC and the posterior inferior temporal areas (including the EBA), which probably signify the suitability and sensitivity of task-dependent processing [21,66]. This phenomenon occurs during the memory maintenance period following the disappearance of stimuli, enabling observers to interpret their emotional significance and reenact postural features. Therefore, we infer that maintaining the connections during this phase is crucial for VWM related to processing bodily postures. One limitation is our failure to differentiate whether these connections reflect information processing related to body postures or their semantic representation. It is plausible that both processes are involved, warranting further investigation to delineate potential distinct stages.

### 4.4. Deep Exploration of Abstract Concepts Beyond Simple Repetitions of Information

The observed connections in the results provide potential support for their role in the rehearsal of body representations. This is consistent with findings that the insula, PFC, and pre-SMA are crucial components of the fronto-parietal network, exhibiting co-activation in memory control [3,67]. However, multivariate analyses revealed significant results in the left anterior temporal lobe (ATL). The enhanced decoding accuracy for happy stimuli compared to neutral ones within the ATL suggests a degree of semantic comprehension related to emotional concepts. The ATL is widely recognized as a key region for semantic control, posited to function as a “transmodal” hub integrating semantic features across various sensorimotor systems and language processing areas [68,69]. Recent findings further indicate a functional network between the prefrontal regions and temporal lobe, which modulates the information stream for semantic understanding [18,70]. This supports our hypothesis that interactions from perceptual areas such as the EBA to the PFC may also play a significant role in semantic comprehension. Additionally, our recent work aims to elucidate early processing patterns of emotional concepts [71], consistent with current designs suggesting that conceptual processing begins approximately 300 ms after stimulus presentation.

### 4.5. Limitations

We used different comparable analyses to provide neuroimaging evidence for the VWM processing of bodily expression. However, there are several limitations in this study as follows: 1. Incorporating methods such as electroencephalography (EEG) could provide complementary evidence. EEG has a higher temporal resolution compared to fMRI [72]. 2. Generalization to other emotions does not imply analogous processing across different brain regions. Forced-choice expressions, though controlled, may not accurately represent real-world scenarios where emotional perception occurs spontaneously. 3. By concentrating on regions such as the EBA, FBA, and PFC, the current study may overlook other brain areas that could play a supportive or indirect role. It would be better to consider a whole-brain analysis, such as the large-scale brain network methodology [73], to capture additional VWM-relevant regions.

## 5. Conclusions

The primary objective of the current study was to investigate how emotional types of bodily expressions influenced brain activation and neural representations, as well as to explore whether the corresponding areas would show a functional coupling among the initial processing regions. Overall, current findings suggest that information storage plays a significant role in processing emotional bodies. The emotional effects also highlight that bodily posture may extend interactions to a broader brain network context within the fronto-temporal network.

## Figures and Tables

**Table 1:** Group-level statistics in univariate and multivariate results. The table below shows two groups of regions, those with significant main effects of emotion in the univariate results and the others for inter-emotion decoding in the multivariate results.

	MNI Coordinates (Peak)				
Brain Areas	x	y	z	*F* or *t*	Cluster Size
Main effects of emotion					
PFC (IFG/MFG/AI)	−46	32	8	20.951	980
PFC (IFG/AI)	50	24	12	15.155	349
AI	40	30	−8	11.571	63
ACC (mPFC)	6	54	−10	11.887	344
pre-SMA	−6	22	42	10.954	169
IPS	−32	−42	40	12.836	89
AG	52	−52	26	10.443	31
EBA	−50	−56	−2	23.077	649
Inter-emotion decoding					
PFC (IFG/MFG)	−48	26	28	7.198	765
PFC (IFG/MFG)	54	18	26	4.867	88
ACC	6	34	24	4.715	54
PreC	−32	−20	64	6.285	139
IPS	−32	−60	46	5.463	100
Precu	2	−72	30	6.294	223
EBA	−54	−62	−2	6.493	235

**Table 2:** Group-level pairwise comparison statistics in univariate and multivariate results. It presents four groups of regions, those with significant activations for happiness vs. neutral and anger vs. neutral in univariate results and those with higher decoding accuracy for happiness vs. neutral and anger vs. neutral in multivariate results.

	MNI Coordinates (Peak)				
Brain Areas	x	y	z	*t*	Cluster Size
Univariate Analysis					
Happiness > Neutral					
PFC(IFG/MFG/AI)	50	24	12	5.488	1451
PFC(IFG/AI)	−46	30	8	5.956	1733
pre-SMA	−6	22	44	4.382	358
EBA(FBA)	−50	−56	−4	6.85	1078
EBA	50	−60	−10	4.61	135
IPS	−32	−42	38	4.861	240
Anger > Neutral					
PFC(IFG/AI)	−42	24	8	5.427	450
PFC(IFG)	54	32	4	4.93	120
EBA	−48	−60	−4	4.31	68
Multivariate Analysis					
Happiness > Neutral					
ATL	−50	−4	−30	4.939	66
PFC(MFG/IFG)	−38	22	20	6.732	342
PFC(MFG)	−46	12	42	5.16	88
PFC(WM)	44	32	28	5.641	62
CS	−34	−24	60	6.594	340
IPS	−44	−42	40	6.184	847
IPS	26	−70	30	6.292	433
SOG	−32	−80	24	5.728	76
pre-SMA	−6	28	46	5.197	74
MCC	4	−30	36	6.13	239
PreC	−36	−2	46	4.504	57
PreC	38	−4	44	4.721	55
EBA(FBA)	−48	−62	−4	6.265	627
EBA	48	−56	−6	4.878	53
Anger > Neutral					
PFC(IFG/MFG)	52	16	28	5.925	31
PFC(MFG)	36	56	16	7.207	62
EBA	−56	−66	−4	6.205	80

## Data Availability

The data presented in this study are available upon request from the corresponding author due to the data containing information that could compromise the privacy of research participants.
